# Intracranial Pressure Monitoring and Treatment Thresholds in Acute Neural Injury: A Narrative Review of the Historical Achievements, Current State, and Future Perspectives

**DOI:** 10.1089/neur.2023.0031

**Published:** 2023-08-07

**Authors:** Kevin Y. Stein, Logan Froese, Alwyn Gomez, Amanjyot Singh Sainbhi, Nuray Vakitbilir, Younis Ibrahim, Frederick A. Zeiler

**Affiliations:** ^1^ Biomedical Engineering, Price Faculty of Engineering, Rady Faculty of Health Sciences, University of Manitoba, Winnipeg, Manitoba, Canada.; ^2^ Section of Neurosurgery, Department of Surgery, Rady Faculty of Health Sciences, University of Manitoba, Winnipeg, Manitoba, Canada.; ^3^ Department of Human Anatomy and Cell Science, Rady Faculty of Health Sciences, University of Manitoba, Winnipeg, Manitoba, Canada.; ^4^ Department of Clinical Neuroscience, Karolinska Institutet, Stockholm, Sweden.; ^5^ Division of Anaesthesia, Department of Medicine, Addenbrooke's Hospital, University of Cambridge, Cambridge, United Kingdom.

**Keywords:** acute traumatic neural injury, ICP monitoring, ICP thresholds, TBI

## Abstract

Since its introduction in the 1960s, intracranial pressure (ICP) monitoring has become an indispensable tool in neurocritical care practice and a key component of the management of moderate/severe traumatic brain injury (TBI). The primary utility of ICP monitoring is to guide therapeutic interventions aimed at maintaining physiological ICP and preventing intracranial hypertension. The rationale for such ICP maintenance is to prevent secondary brain injury arising from brain herniation and inadequate cerebral blood flow. There exists a large body of evidence indicating that elevated ICP is associated with mortality and that aggressive ICP control protocols improve outcomes in severe TBI patients. Therefore, current management guidelines recommend a cerebral perfusion pressure (CPP) target range of 60–70 mm Hg and an ICP threshold of >20 or >22 mm Hg, beyond which therapeutic intervention should be initiated. Though our ability to achieve these thresholds has drastically improved over the past decades, there has been little to no change in the mortality and morbidity associated with moderate-severe TBI. This is a result of the “one treatment fits all” dogma of current guideline-based care that fails to take individual phenotype into account. The way forward in moderate-severe TBI care is through the development of continuously derived individualized ICP thresholds. This narrative review covers the topic of ICP monitoring in TBI care, including historical context/achievements, current monitoring technologies and indications, treatment methods, associations with patient outcome and multi-modal cerebral physiology, present controversies surrounding treatment thresholds, and future perspectives on personalized approaches to ICP-directed therapy.

## Introduction

Since its inception, intracranial pressure (ICP) monitoring has established itself as a fundamental pillar of neurocritical care practice. Persistently elevated ICP can have disastrous effects on the cerebral environment, secondary to brain herniation or insufficient cerebral blood flow (CBF). The latter can be illustrated by the inherent relationship that exists between ICP, cerebral perfusion pressure (CPP), and mean arterial pressure (MAP); CPP = MAP – ICP. It has been shown that such elevations in ICP are associated with poorer outcomes,^1–10^ and that aggressive ICP control results in improved recovery in severe traumatic brain injury (TBI)-injured patients.^1,9,11,12^ Therefore, therapeutic intervention aimed at preventing intracranial hypertension (ICH) is essential for the management of moderate/severe TBI. However, because of the notable risks of ICP-lowering therapeutics, clinicians must balance the danger of elevated ICP with the iatrogenic risks of such therapies. Therefore, accurate ICP monitoring, that is safe and simple to use, is necessary to guide ICP maintenance.

Current management guidelines now include ICP thresholds, >20 or >22 mm Hg, beyond which therapeutic intervention is recommended.^13,14^ Despite major improvements in our ability to achieve such thresholds over the past few decades, there has been very little change in the morbidity and mortality associated with severe TBI.^15–17^ This is, in part, attributable to a failure to consider individual phenotype, given that cerebral physiological response to TBI varies greatly between persons.^18–23^ A potential solution to this problem is through the development of individualized ICP thresholds that are patient specific and continuously derived.

In this narrative review, we will begin by providing a quick historical overview of the development of ICP monitoring. We will then discuss ICP monitoring technologies, indications for their use, and therapeutic approaches for controlling ICP. We will also examine treatment thresholds and their associations with outcome and multi-modal cerebral physiology. Finally, we will conclude with a discussion of the controversies surrounding the use of these thresholds and future directions in ICP-directed care.

## History of Intracranial Pressure and the Monro-Kellie Doctrine

The concept of ICP—the pressure created by the contents of the cranium—was first introduced in 1783 when Alexander Monro described the cranium as a rigid compartment containing a “nearly incompressible” brain, and concluded that intracranial blood volume must therefore remain constant.^24–27^ In 1824, a former student of Monro, George Kellie, provided support for this hypothesis by demonstrating that intracranial blood volume was relatively similar between autopsies, regardless of the cause of death.^27,28^ Based on this observation, Kellie asserted that in order for total intracranial blood volume to remain constant, decreases in arterial blood volume must be compensated for by engorgement of the venous system.^28^ The work of these two Scotsmen would set the framework for what would later become known as the Monro-Kellie doctrine. A few years later, another Scottish surgeon, John Abercrombie, further supported this growing hypothesis by demonstrating that, as long as the skull and dura remain intact, the brain does not exsanguinate like other organs post-mortem.^27,29^ This indicated that the cranium was a closed system with a relatively negative internal pressure.

Up until this point, the developing doctrine only considered the presence of blood and brain tissue; however, this changed when the French physiologist, François Magendie, described cerebrospinal fluid (CSF) circulation, and the English physician, George Burrows, incorporated its role into the hypothesis.^27,30–32^ However, the so-called Monro-Kellie doctrine, as it is known today, was not fully synthesized until the renowned American neurosurgeon, Harvey Cushing, compiled the various contributions into a succinct synopsis, which, to this day, serves as a fundamental concept in the field of neurosurgery.^27,33,34^

The modern doctrine states that, with an intact skull and dura, the cranial compartment is of fixed volume and that the combined intracranial volume of brain parenchyma, blood, and CSF must remain constant, and therefore an increase in the volume of one component must be offset by a decrease in the volume of another component.^32,33^ This doctrine lays out the fundamental principles that dictate ICP dynamics and is crucial for understanding the detrimental effects of ICH.^32,34^ It should be noted that this concept does not apply to the infant given that the sutures of the infantile skull have not yet fused and thus allow for some degree of volumetric compliance.^35^

Given that the contents of the cranium are relatively incompressible, stable ICP requires a volumetric equilibrium, where changes in the volume of one component are compensated for by changes in the volume of the other two components.^24,34^ This compensatory reserve is primarily provided by the brain's venous blood pool, which can be adjusted to maintain stable ICP.^34^ The brain's CSF pool can also contribute to the maintenance of physiological ICP, however, to a lesser extent.^27,34^ When a pathological increase in intracranial volume occurs, the initial increase is readily compensated for by the movement of venous blood and CSF out of the cranium; however, this mechanism is limited and can quickly become depleted.^24^ Once this point of decompensation is reached, even a miniscule elevation in volume can result in substantial increases in ICP.^27^ The resulting ICH can have devastating effects on the intracranial environment, such as cerebral ischemia and brain herniation, both of which can cause irreversible brain damage.^25,34^ Such exhaustion of the compensatory reserve occurs in a wide range of pathological states, such as TBI and intracranial space occupying lesions or bleeds.^24^ Therefore, maintaining physiological ICP, generally defined as ∼5–15 mm Hg in the healthy supine adult,^24,25^ is critical for limiting further brain injury in such patients, with accurate monitoring of ICP necessary to guide clinical management and prognostication.^34^

## Origins of Intracranial Pressure Monitoring

In 1916, the German neurologist, Hans Queckenstedt, made the first attempts at measuring intraspinal pressure when he performed lumber punctures and approximated pressure using U-tube manometry.^36^ However, it was not until the early 1950s when the first true ICP measurement was taken. It was two Frenchmen, Jean Guillaume and Pierre Janny, who used an external ventricular drain (EVD), which involves placing a catheter into the anterior horn of a lateral ventricle to allow CSF to flow externally, and a U-tube manometer to measure ICP.^36–39^ This laid the framework for the development and implementation of modern ICP monitoring. A decade later, the Swedish neurosurgeon, Nils Lundberg, developed an improved monitoring technique that facilitated the continuous measurement of ICP.^36^ His technique utilized a strain-gauge pressure transducer and a paper recorder, which allowed for temporal observations of ICP and characterization of its waveform.^36,39,40^ He continued on to establish protocol for its use and demonstrate its efficacy and safety; however, despite this, there was widespread skepticism surrounding its utility and safety among the neurointensive care community.^36^

For the most part, ICP monitoring remained primarily a research tool until the late 1970s and 1980s, when its use finally began gaining acceptance because of growing evidence, indicating that aggressive ICP maintenance resulted in improved outcomes.^24,36^ At the turn of the century, ICP monitoring was finally incorporated into standard neurocritical care practice when its use was included in the U.S. Brain Trauma Foundation (BTF) guidelines.^1,41^ Today, ICP monitoring is considered a cornerstone of neurocritical care as an indispensable tool for guiding the therapeutic management of TBI.^38^

## Current Intracranial Pressure Monitoring Devices

### External ventricular drain

Since the introduction of ICP monitoring using the EVD, various other methods for monitoring ICP have been developed and popularized. However, intraventricular pressure monitoring through the use of an EVD remains the gold standard of ICP monitoring.^34^ This method involves inserting a catheter directly into a ventricle, usually through a coronal burr hole.^24,25^ Currently, the catheter is connected to an external strain-gauge pressure transducer, whose electrical resistance varies depending on the extent it is deformed by the pressure of the surrounding environment.^34,42^ The electrical resistance then dictates the voltage of the device's internal circuitry, thus producing pressure measurements.^42^ A major advantage of this setup is that it allows for recalibration of the device at any time by resetting the device to atmospheric pressure.^24,25^ The EVD is also highly reliable and allows for intrathecal administration of drugs and therapeutic drainage of CSF.^24,25^ However, this device's practicality is hindered by its highly invasive nature and its association with infectious and hemorrhagic complications, 5–20%^43,44^ and 2–10%,^45,46^ respectively.^34^ Additionally, EVD placement may be difficult in cases involving small or compressed ventricles.^24,25^

### Microtransducer devices

A newer invasive method for monitoring ICP is the use of implantable microtransducers, which include fiber optic devices, strain gauge devices, and pneumatic sensors.^25^

In fiber optic devices, such as the Camino ICP Monitor, variations in ICP displace a mirror that alters the intensity of light which is reflected back to a fiber optic cable.^25,34^ A diaphragm and a microprocessor amplifier are then used to detect changes in the intensity of the reflected light to produce an ICP measurement and its waveform.^24^ Though the associated risk of infection or hemorrhage is nearly negligible, this method is relatively expensive, can be quite meticulous to set up, and commonly demonstrates a baseline drift.^24^

With strain gauge devices, such as the Codman MicroSensor, the Raumedic Neurovent-P ICP sensor, and the Pressio sensor, changes in ICP cause a diaphragm within the sensor to bend, resulting in changes in its electrical resistance and thus allowing for ICP measurement.^24,25,34^ These devices have been extensively studied and have been demonstrated to be highly accurate and negligibly associated with infectious and hemorrhagic complications.^24^ However, similar to fiber optic devices, baseline drift is a significant problem in these devices.^24^

Pneumatic sensors, such as the Spielberg ICP Monitor, use a small balloon at the tip of a probe, on which the pressure of the surrounding tissue is exerted, allowing them to produce ICP measurements.^24,25,34^ These devices are becoming increasingly popular because of recent innovations that allow for CSF drainage and automated drift corrections.^24^

Microtransducer devices are most often placed intraparenchymal, usually in the right frontal cortex; however, they can also be placed in the subarachnoid, subdural, or epidural spaces.^24,34^ These devices provide a less invasive alternative to the EVD that are more simple to place, are associated with lower infection and hemorrhage risk, and have a lower incidence of waveform damping and signal artifacts, while at the same time maintaining a close correlation to the gold-standard measurements.^24^ However, most microtransducers suffer from the inability to recalibrate, and thus the problem of baseline drift, as well as increased cost.^24,34^

### The problem of baseline drift

As discussed above, a major shortcoming of most microtransducer devices is the problem of baseline drift, also known as zero drift. Baseline drift describes the phenomenon where a sensor reports increasingly inaccurate measurements over time, resulting from a lack of continuous calibration.^25^ The magnitude of baseline drift can be determined by looking at the ICP value that is measured when a device is removed versus when it was placed.^25^ The problem of baseline drift presents a clear concern for patient care, given that proper treatment requires accurate ICP measurements to guide management. The exact mechanism behind this phenomenon, in regard to ICP monitoring devices, remains unclear. For a thorough overview of the accuracy of various ICP monitoring devices and the extent of baseline drift in these devices, we refer the reader to an article by Pelah and colleagues.^47^

### Non-invasive modalities

Though invasive methods of measuring ICP are accurate and reliable, their invasive nature and risk of complications have driven research studying potential non-invasive alternatives.^24^ Such non-invasive methods should, in theory, eliminate infectious and hemorrhagic complications, allow for safe screening of low-risk persons, be more simple and convenient to use, be readily available, and be relatively inexpensive.^24,34^ Dozens of promising non-invasive technologies exist, including transcranial Doppler, transocular ultrasonography measuring optic nerve sheath diameter, and near infrared spectroscopy.^24,25,34^ Despite the great strides that have been taken in the development of non-invasive ICP monitors, invasive devices remain to be the most accurate methods to measure ICP, with EVDs and intraparenchymal strain gauge devices being the most commonly used in clinical practice.^24^ The various invasive and non-invasive monitoring modalities are summarized in Table 1.

**Table 1.** Summary of ICP Monitoring Methods

***Monitoring method***	***Placement location***	***Mechanism***	***Pros***	***Cons***
External ventricular drain	Ventricle	CSF is allowed to drain to an external pressure transducer.^24^	- Highly accurate (gold standard) - Can be recalibrated - Allows for intrathecal administration of drugs - Allows for CSF drainage	- Invasive - Infection and hemorrhage risk - Can be challenging to place in certain situations
Strain gauge	Usually, in the parenchyma	Variations in ICP cause a diaphragm within the sensor to bend.^24^	- Highly accurate - Simple to place	- Invasive - Baseline drift
Fiber optic	Usually, in the parenchyma	Variations in ICP displace a mirror that alters the intensity of light which is reflected back to a fiber optic cable.^24^	- Highly accurate	- Invasive - Baseline drift - Challenging to place - Expensive
Pneumatic sensors	Usually, in the parenchyma	Pressure is exerted onto a small balloon at the tip of a probe.^24^	- Highly accurate - Can be recalibrated - Allows for CSF drainage	- Invasive
Transcranial Doppler	Non-invasive	Estimates ICP by measuring the velocity of blood flow in the middle cerebral artery.^38^	- Non-invasive	- Mixed evidence on its efficacy to estimate ICP - Interoperator variability
ONSD	Non-invasive	Because there is dura overlying the optic nerve, increased ICP results in an expansion of the subarachnoid space around the nerve, thus increasing the diameter of the nerve sheath.^38^	- Non-invasive	- Mixed evidence on its efficacy to estimate ICP - Interoperator variability - Normal ONSD varies between persons

ICP, intracranial pressure; CSF, cerebrospinal fluid; ONSD, optic nerve sheath diameter.

## Indications for Intracranial Pressure Monitoring

The BTF guidelines recommend the use of ICP monitoring for certain severe TBI-injured patients.^1,13^ However, the lack of a supporting randomized control trial prevented its establishment as a standard of care early on.^1^ Given the invasive nature of ICP monitoring and its associated risks, its use should be limited to patients who are at high risk of ICH. Multiple studies identified comatose patients, those with a Glasgow Coma Scale (GCS) score of ≤8, as a high-risk population.^1–3,11^ Additionally, Narayan and colleagues found that, within this population, those with an abnormal computerized tomography (CT) scan had a significantly higher incidence of ICH than those with a normal scan, ∼60% and 13%, respectively.^11^ Other studies demonstrated similar findings.^12,48^ However, patients with normal CT scans tended to demonstrate similar incidence rates as those with abnormal scans when they presented with at least two of the following adverse features: age >40 years, motor posturing, and systolic blood pressure <90 mm Hg.^11^ Given that symptoms of elevated ICP, such as headache, are not usually identifiable in a comatose patient, and clinical signs, such as papilledema, do not consistently present with ICH, ICP monitoring is necessary in such high-risk populations.^49^

Based on these findings, the BTF guidelines recommend initiation of ICP monitoring in patients with severe TBI, defined as a GCS of 3–8, with an abnormal CT scan or at least two of the adverse features detailed above.^1,13^ However, it should be noted that most of these findings are from studies that were done over four decades ago and that CT technology has significantly improved since then. Monitoring in patients with mild or moderate TBI is not routinely recommended, because of its invasive nature and the relatively low risk of ICH in this population.^1,13^ However, clinicians may consider monitoring in this population in certain scenarios, such as when treatment that prevents proper serial neurological examinations or can cause elevations in ICP is required.^49^ ICP monitoring is usually continued until ICP remains normal for a prolonged period of time, usually 24–48 h, without ICP-lowering therapy.^49^ Unfortunately, despite the recommendations of the BTF, up to half of patients who are indicated for ICP monitoring do not receive it.^50,51^

The only contraindication for ICP monitoring is the presence of a coagulopathy.^52^ It should be noted that this is considered a relative contraindication that clinicians may choose to disregard in certain situations based on clinical judgement. In the coagulopathic patient, the placement of an ICP monitor or EVD is preferably avoided until the coagulopathy can be reversed; however, in emergent cases, placement can be performed in tandem with correction. Placement of an ICP monitor is rarely considered an emergent procedure and will usually be deferred until after reversal. On the other hand, EVDs are occasionally used for aggressive ICP reduction, and can be placed regardless of the presence of a coagulopathy.

## Intracranial Pressure Waveform

As discussed earlier, a key protection mechanism is the ability of the craniospinal system to compensate for volumetric changes. Exhaustion of this craniospinal compliance can rapidly result in injury to the brain and is therefore important to monitor. Though ICP level itself can give some insight into the state of this compliance, it usually is unable to identify impaired compliance in time to prevent its detrimental effects. The shape of the ICP waveform can give further insight into the state of craniospinal compliance and help detect pathologies earlier. ^53,54^ Additionally, monitoring the ICP waveform shape can assist in evaluating the effectiveness of interventions aimed at improving craniospinal compliance. For example, after initiating treatment for elevated ICP, such as administering osmotic agents, changes in the waveform shape can indicate the response to treatment and guide further management decisions.

The ICP waveform represents the dynamic changes in ICP. When measured continuously, ICP demonstrates a pulsatile waveform made up of a waveform corresponding to the respiratory cycle superimposed onto a waveform corresponding to the cardiac cycle.^24^ However, the prominent saw-tooth pattern pulsations are primarily driven by the cardiac cycle. The shape of the ICP waveform is influenced by several factors, including the compliance of brain tissue, CSF production, absorption, and circulation dynamics.

The aspect of the ICP waveform that has been most looked at is the fundamental amplitude of the pulse waveform (AMP). Usually, increases in ICP are associated with increases in AMP; however, at very high levels of ICP, AMP begins to decrease. This is because when ICP becomes very high, the pressure on the vasculature exceeds diastolic ABP, causing cerebrovascular collapse and cessation of diastolic flow.^55^ This ICP level at which AMP begins to decrease instead of increase is known as the upper breakpoint of amplitude-pressure characteristic.^55^ Increased AMP is often considered a sign of poor craniospinal compliance given that it indicates that there are larger changes in pressure in response to changes in volume (blood flow). Further, it has been shown that elevated AMP is associated with poorer outcome in TBI.^56^

Another important measure that can be derived from waveform analysis is the Pearson correlation between AMP and ICP, termed the RAP index.^57^ This index is a measure of compensatory reserve and can be used to estimate craniospinal compliance. A RAP value near 0 indicates that variation in AMP is not driven by changes in ICP, thus suggesting intact craniospinal compliance, whereas values near 1 indicate that AMP changes are being driven by ICP changes and that there is a lack of compliance. Additional waveform analysis techniques exist, such as the analysis of waveform patterns or measurement of the slope of pulsations; however, a full overview of these techniques is not included here.

## Intracranial Pressure–Lowering Therapeutics: Current Treatment Paradigm

The primary utility of ICP monitoring is to guide therapeutic management aimed at maintaining physiological ICP and preventing ICH. In the supine adult, normal ICP is loosely defined as ICP of 5–15 mm Hg, with mild ICH generally described as 20–30 mm Hg and severe ICH characterized as sustained ICP above >40 mm Hg.^49^ However, it should be noted that baseline ICP levels can vary greatly between persons. For example, a 2012 study by Berdahl and colleagues found a direct linear relationship between body mass index (BMI) and mean CSF pressure (*R*^2^ = 0.2, *p* < 0.001), with mean pressure increasing from 8.6 ± 2.1 mm Hg for a BMI of 18.0–14.1 ± 2.5 mm Hg for a BMI of 39.^58^ They also noted that this elevation in mean CSF pressure was not necessarily associated with poorer outcome, thus indicating that physiological ICP may be higher in patients who are obese.^58^ Therefore, such patient-specific factors should be considered when determining baseline levels and deciding whether initiation of ICP-lowering therapeutics is warranted.

The goal of maintaining physiological levels of ICP is to prevent secondary brain insults caused by brain herniation and insufficient CBF.^24^ CPP, ICP, and MAP are inherently related through the following formula: CPP = MAP – ICP.^24^ MAP is defined as the average arterial blood pressure (ABP) over one cardiac cycle and is typically acquired by indwelling arterial lines for continuous blood pressure acquisition, with zeroing, for the purposes of ICP/CPP-directed management, suggested at the level of the tragus by recent expert consensus statements.^59,60^ Normally, consistent CBF can be maintained over a range of CPP values attributable to the brain's inherent autoregulatory mechanism.^61–63^ However, this mechanism is limited and is often impaired in TBI-injured patients, exposing the brain to pressure-passive insults.^61,62,64^ Therefore, preventing ICH is essential for not only preventing herniation of brain tissue, but also maintaining sufficient CPP to adequately perfuse cerebral tissue and prevent ischemia.

**FIG. 1.**
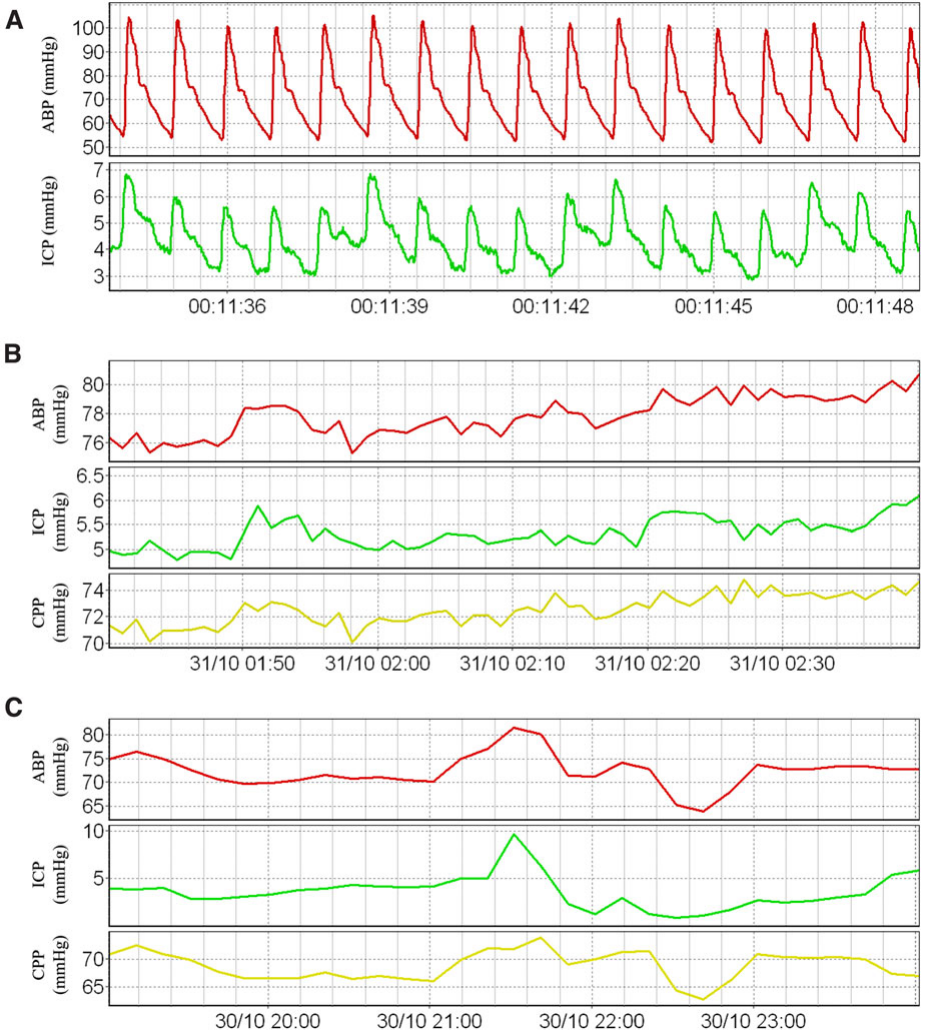
Patient example of cerebral physiological data recordings. (**A**) High-resolution pulse waveform data recordings of ABP and ICP. (**B**) Data recordings of ABP, ICP, and CPP with a 1-min smoothing average filter applied. (**C**) Data recordings of ABP, ICP, and CPP with a 10-min smoothing average filter applied. ABP, arterial blood pressure; CPP, cerebral perfusion pressure; ICP, intracranial pressure. Data from Winnipeg Acute TBI Laboratory Database, with full ethics approval in place for access, processing, and publication (H2017:181, H2017:188, H2020:118).

Management guidelines recommend initiation of ICP-lowering therapies when CPP drops below 60 mm Hg or ICP surpasses 20 or 22 mm Hg.^13,14,49^ It should be noted that, though ICP should remain relatively stable over time in the normal state, it does not remain perfectly constant. As mentioned earlier, ICP demonstrates a pulsatile waveform made up of a waveform correlating with the respiratory cycle superimposed onto a waveform correlating with the cardiac cycle.^24^ Regular fluctuations in ICP are also expected during postural changes and with variations in adrenergic tone.^34,35^ Therefore, in the clinical setting, time-averaged ICP is usually used for establishing baseline values and only prolonged elevations in ICP are considered clinically significant.^34^ A patient example of raw cerebral physiological data and time-averaged recordings is presented in Figure 1.

General care for ICH involves preventing and treating factors that exuberate elevations in ICP, such as obstruction of venous return, respiratory failure, hypertension, hyponatremia, anemia, fever, and seizures.^49^ When ICP is elevated for prolonged periods of time, additional therapeutic interventions are required to control ICP.

**FIG. 2.**
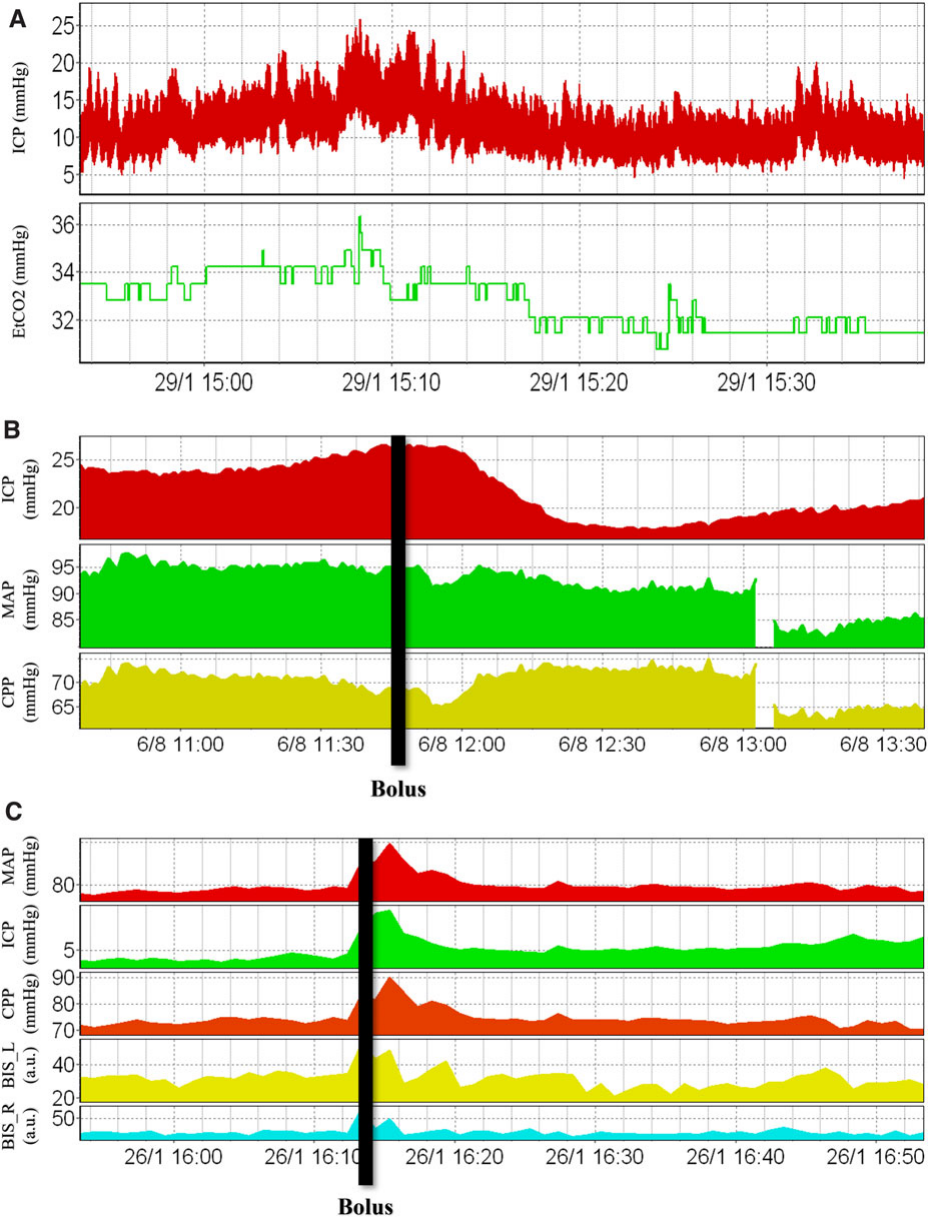
Cerebral physiological responses to ICP-lowering therapeutics. (**A**) Association between ICP and EtCO_2_. (**B**) ICP, MAP, and CPP responses to a bolus dose of hypertonic saline. (C) ICP, MAP, CPP, and BIS left/right responses to a bolus dose of sedation. a.u., arbitrary units; BIS, bispectral index; CPP, cerebral perfusion pressure; EtCO_2_, end-tidal carbon dioxide; ICP, intracranial pressure; MAP, mean arterial pressure. Data from Winnipeg Acute TBI Laboratory Database, with full ethics approval in place for access, processing, and publication (H2017:181, H2017:188, H2020:118).

A commonly used method for reducing ICP is hyperventilation. This therapeutic approach decreases the partial pressure of carbon dioxide (CO_2_) of blood, resulting in constriction of cerebral vessels and a reduction of cerebral blood volume, thus reducing ICP.^49^ However, hyperventilation is of limited utility because of the short-term nature of its effects, potential for rebound hyperemia, and risk of cerebral ischemia.^49^ In a randomized control study by Muizelaar and colleagues, it was demonstrated that prolonged hyperventilation leads to poorer outcomes in severe TBI-injured patients by precipitating ischemic episodes.^65^ Therefore, its use is largely restricted to the acute treatment of elevated ICP, allowing time for other ICP-lowering therapies to be initiated.^49^ Because hyperventilation accelerates CO_2_ elimination, end-tidal CO_2_ (EtCO_2_) is closely related to the extent of hyperventilation and is therefore used for titrating this therapy. A patient example of the association between ICP and EtCO_2_ can be found in Figure 2A.

Another therapeutic option for lowering ICP is hyperosmolar agents, of which mannitol and hypertonic saline are the most popular. These agents quickly act to lower ICP by increasing plasma volume, which induces constriction of cerebral vasculature, as well as by increasing serum osmolarity, which draws fluid out of the cerebral parenchyma.^49^ However, these agents have various potential side effects, such as hypovolemia, hyperosmolarity, and renal failure.^49^ Further, a study by Kaufmann and Cardoso showed that repeated doses of such agents can even perpetuate brain swelling.^66^ Additionally, these agents induce diuresis, thus necessitating fluid replacement, and require tapering when stopped in order to avoid rebound elevations in ICP.^49^ A patient example of the responses of ICP, MAP, and CPP to a bolus dose of hypertonic saline can be found in Figure 2B. Over the past years, hypertonic saline has become the preferred hyperosmolar agent; however, its effectiveness compared with other agents, such as mannitol, is still highly contended. A 2019 Cochrane meta-analysis suggested that hypertonic saline is not superior to mannitol, in terms of efficacy and safety, for the acute management of TBI.^67^ However, other more recent meta-analyses have suggested that hypertonic saline is associated with lower treatment failure and more sustained effects on ICP.^68,69^

Sedatives, analgesics, and paralytics are routinely used in neurocritical care practice to prevent agitation and elevations in blood pressure, both of which can cause increases in ICP.^1^ An obvious disadvantage of using such agents is that they complicate the interpretation of the neurological examination.^1^ In extreme cases of unresponsive ICH, barbiturate coma has been shown to improve outcome,^70^ with a randomized control trial by Eisenberg and colleagues demonstrating a 2-fold increase in the probability of controlling ICP.^4,49^ However, barbiturates are associated with significant complications, such as hypotension, hypokalemia, respiratory failure, infection, and organ dysfunction, as well as preventing the interpretation of the neurological examination for several days.^49^ Therefore, ICP monitoring is necessary for deciding when the benefits of initiating such therapy outweigh the risks and when it should be discountinued.^1^ A patient example of the responses of ICP, MAP, CPP, and bispectral index (BIS), an indicator of the depth of sedation, to a bolus dose of a sedative can be found in Figure 2C.

CSF drainage, with the use of an EVD, is an effective method for quickly and dramatically reducing ICP.^49^ However, its use is limited in that overly aggressive drainage can result in ventricular collapse with loss of ICP transduction and that it fails to tackle the underlying pathophysiology present in TBI, which is rarely hydrocephalus. Therefore, although a popular therapeutic method historically, there has been a shift away from its use for managing trauma-related ICH. Currently, some clinicians still elect to use EVDs because of their ability to rapidly reduce and monitor ICP while more escalations in surgical or medical management are arranged; however, with the improvements in microtransducer ICP monitoring devices and the safety profiles of other therapeutic options, this pattern is likely to continue.

Additional ICP-lowering therapies exist, such as induced hypothermia, administration of steroids, and surgical intervention, which can be used in conjunction with the therapeutics described above.^49^ Recent clinical trials indicate that induced hypothermia results in lower ICP, but has no beneficial impact on clinical outcome.^49,71,72^ Steroids have been demonstrated to decrease ICP in brain tumor patients; however, for other pathologies, such as TBI, steroids have been shown to have detrimental effects and therefore should not be used.^49,73^ Emergent surgical procedures, such as mass resection and decompressive craniectomy, are utilized when ICH occurs rapidly or is unresponsive to medical therapeutics.^49^ It should be noted that all therapeutic options for reducing ICP demonstrate notable risks; therefore, the clinician must balance the threat of ICH with the iatrogenic risks of such therapies.^1^

## Association Between Intracranial Pressure Control and Outcome

At the time of the development of the BTF guidelines, there existed an extensive body of literature that indicated that higher ICP was associated with poorer outcomes.^1–10^ There was also evidence that ICP monitoring could contribute to more accurate outcome prognostication than the use of solely clinical exaination.^2,10^ Further, studies had demonstrated that ICP control increases the probability of recovery, by reducing the risk of brain herniation and cerebral ischemia.^1,9,11,12^ A 1977 study by Becker and colleagues reported a reduction in mortality rate when an intensive management protocol that utilized ICP monitoring was included into patient care.^5^ Similarly, a prospective placebo-controlled study by Eisenberg and colleagues also reported improved outcome when ICP was controlled.^4^ Ghajar and colleagues compared a group of patients who had their ICP monitored and were treated for elevated ICP versus a group who did not. They found that the mortality rate of the monitored group was significantly lower than the non-monitored group, 12% and 53%, respectively.^74^ It had also been shown that systemic hypotension was associated with poorer outcome, likely as a result of inadequate cerebral perfusion attributable to the direct relationship that exists between blood pressure and CPP.^1,75^ In a study by Marmarou and colleagues, it was shown that the proportions of time with ICP above 20 mm Hg and blood pressure below 80 mm Hg were highly predictive of outcome.^2^

Recent literature has affirmed many of the above-described findings, with a multitude of studies demonstrating that aggressive ICP maintenance is associated with improved outcome.^51,76,77^ Additionally, recent work has described a strong relationship between elevated ICP and mortality in severe TBI.^78–84^ The association between ICP and favorable outcome has also been validated; however, it has been found to be less robust than that of mortality.^78,80,81^ Interestingly, the only randomized control trial investigating ICP monitoring to date, by Chestnut and colleagues, found that management directed using ICP monitoring did not result in statistically superior outcomes when compared to management based solely on clinical examination and imaging.^36,85^ This study, which took place in Bolivia and Ecuador, essentially compared two methods of ICP-directed therapy, one where ICP was monitored directly and the other where ICP was estimated using clinical examination and imaging, and does not comment on the effect of ICP control itself. Therefore, these results do not refute the importance of ICP-based management strategies.

## Association Between Intracranial Pressure Control and Multi-Modal Cerebral Physiology

Multi-modal monitoring refers to the various bedside physiological monitoring modalities that supplement ICP monitoring and provide the clinician with a more complete picture of the patient's cerebral physiological state. These modalities include those that monitor CBF, systemic hemodynamics, brain oxygenation, cerebral autoregulation, cerebral metabolism, and electrophysiology.^20^ A recent systematic review by our group found that there is very limited literature that demonstrates direct statistical associations between such modalities and ICP above/below guideline-defined thresholds.^86^

We were not able to find any studies demonstrating an association between ICP above/below threshold and CBF directly; however, multiple studies were found for CBF velocity. These studies found that CBF velocity had little to no relationship with ICP above/below threshold within the therapeutic range of ICP.^87–91^ For the category of systemic hemodynamics, a study by Zeiler and colleagues found that there was a statistically significant difference in MAP between a patient cohort with a mean ICP of <15 mm Hg and a cohort with a mean of >20, 82, and 89 mm Hg, respectively (*p* = 0.0009).^92^ A study by Mowery and colleagues demonstrated that increasing ranges of ICP were associated with increased cardiac decoupling, with analysis of variance testing producing a *p* value of <0.001.^93^ A study by Narotam and colleagues, looking at parenchymal brain oxygen tension (PbtO_2_), found that PbtO_2_ <20 mm Hg is associated with ICP >20 mm Hg, with chi-square analysis producing a chi-square value of 9.34 (*p* < 0.01).^94^

Surrogate metrics of cerebral autoregulation have also been shown to be associated with ICP above/below threshold. Zeiler and colleagues demonstrated that mean PRx, pulse amplitude index (PAx; correlation between AMP and MAP), and RAC (the correlation [R] between AMP [A] and CPP [C]) all significantly differed between a patient cohort with a mean ICP <15 mm Hg and a cohort with a mean > 20 mm Hg, producing *p* values of <0.001, 0.003, and 0.003, respectively, on Mann-Whitney U testing.^92^ No studies that demonstrated an objective association between ICP above/below threshold and a metric of either electrophysiology or cerebral metabolism were found in the systematic review.^86^

## Intracranial Pressure Thresholds: Current Guidelines, Limitations, and Future Perspectives

### Guideline-based intracranial pressure thresholds

Though there was a significant amount of literature demonstrating the association between ICP and outcome before publication of the BTF guidelines, there remained questions revolving around what to use as a treatment threshold. In 1991, Marmarou and colleagues conducted a study on 428 severe TBI-injured patients, evaluating ICP thresholds, in 5-mm-Hg increments, and their ability to predict 6-month outcome.^2^ Through a logistic regression analysis, they found that the proportion of time with ICP >20 mm Hg correlated the best with outcome.^2^ This result is in keeping with other smaller studies that evaluated threshold values between 15 and 25 mm Hg.^3,11,95–99^ A study by Saul and Ducker compared outcomes between a group of patients who were treated in 1977–1978 using an ICP threshold of 20–25 mm Hg versus a group that was treated in 1979–1980 using a more strict protocol with a threshold of 15 mm Hg.^96^ They found that the mortality rate was significantly lower in the second group, 46% and 28% (*p* < 0.0005), respectively.^96^ This suggests a possible benefit in using a lower ICP threshold; however, differences in time period and treatment protocols between two groups clouds the certainty of any stand-alone effects of lowering the treatment threshold.^95^

Based on these findings, the BTF published an ICP threshold of 20–25 mm Hg, above which ICP-lowering therapies should be initiated.^95^ A 2013 study by Talving and colleagues found that compliance with this guideline resulted in significantly improved outcomes, providing additional support for this threshold.^100^ In 2011, Sorrentino and colleagues performed a chi-square analysis on a cohort of 459 TBI patients to identify critical thresholds for cerebral physiological metrics.^101^ They found that an ICP threshold of 22 mm Hg produced the highest chi-square value for differentiating survival/death and favorable/unfavorable outcome.^101^ However, because of limitations of this study, the researchers recommended against challenging guidelines on the basis of their findings.^101^ Additionally, it seems that ideal threshold depends heavily on population analyzed, with different studies producing varying threshold values. Despite this, the BTF revised their guidelines in 2017, now recommending an ICP treatment threshold of 22 mm Hg.^13^ Additionally, revised guidelines have established a CPP target range of 60–70 mm Hg and now recommend greater focus on this physiological parameter.^13,14^ There have yet to be any randomized control trials comparing ICP thresholds.

It should be noted, however, that patients can suffer brain herniation at ICP levels lower than the above-mentioned thresholds. A study by Marshall and colleagues demonstrated that, among patients with pupillary abnormalities, a clinical sign of ICH, ICP values as low as 18 mm Hg were found.^102^ Thus, patients being monitored for ICP should receive routine neurological examinations and CT scans to affirm ICP values.^1^

A recent concept that has received increasing attention in recent years is time dose of ICP. This metric is defined as the area under the curve (AUC) and above threshold of ICP across a recording period.^103^ The added benefit of this metric over the percent time with ICP above threshold is that it considers both duration and intensity of ICP above threshold. Therefore, it better represents the extent of ICH that a patient experiences and is better able to differentiate patients who had ICP greatly exceeding threshold from those who had ICP minimally exceeding threshold. Multiple studies have demonstrated that this dose of ICP is associated with poor 6-month outcome^83,84,103,104^; however, there is yet to be a consensus on whether it is superior to percent time with ICP above threshold for predicting outcome.

Although earlier studies have focused on controlling ICP, recent work suggests concentrating on CPP control.^105^ Revised guidelines have established a CPP target range of 60–70 mm Hg and an ICP threshold of >22 mm Hg, beyond which therapeutic treatment is recommended.^13,14^

### Shortcomings

Over the past decades, our ability to achieve the above-mentioned guideline-based thresholds has drastically improved; however, there has been little to no change in the mortality and morbidity associated with moderate-severe TBI.^15–17^ It has been suggested that this may be, in part, attributable to a failure to take individual phenotype into account and individualize therapeutic management.^18^ Various studies have shown that individual cerebral physiological responses to TBI are significantly heterogenous.^19––23^ A 2005 study by Czosnyka and colleagues found that older TBI patients had poorer outcomes in comparison to those who were younger.^106^ In 2008, Czosnyka and colleagues found that there were sex-related differences in outcome as well.^107^ They found that females had higher mortality rates, but only in a subgroup of patients <50 years of age.^107^ When comparing males and females who were >50 years of age, there were no differences.^107^ Further, Åkerlund and colleagues were able to identify six distinct TBI endotypes based on demographic, physiological, biochemical, and clinical data, and found that consideration of such endotypes significantly improved prognostic modeling.^108^

There is also an extensive body of literature on the role that genetic polymorphisms play in TBI outcome.^109^ Various candidate genes affiliated with neurodegeneration, neural repair, blood–brain barrier integrity, inflammatory responses, and neurotransmission have been connected to TBI.^19^ For example, certain polymorphisms in the APOE gene have been shown to be associated with worse outcome in moderate/severe TBI.^110^ We refer the interested reader to multiple reviews covering the various genetic polymorphisms that have been investigated in relation to TBI.^109,111,112^

Recent literature has also demonstrated that a significant portion of cerebral insult burden in moderate/severe TBI is impervious to guideline-based therapeutic interventions and that the effects of such interventions vary between different patient subgroups.^19,113––116^ This problem also extends to prognostication, with studies demonstrating that current population-based models accounts for less than half of outcome variance.^19,117,118^ Therefore, it is likely that not only do optimal interventions and treatment targets vary between patients, but also vary across each patient's time in the intensive care unit (ICU).

As mentioned previously, a major problem with the literature is that there is a high degree of variability when it comes to ideal treatment thresholds, likely attributable to differences in populations analyzed. This further points to significant variability in individual responses to TBI. Additionally, most studies that have investigated ICP thresholds grouped patients using mean ICP values calculated over entire monitoring periods. This method does not capture the full picture and blunts the effects of extreme values of ICP. All of these shortcomings of current guideline-based management of TBI drastically demand a new approach, one that more accurately captures and incorporates individual-specific factors.

### Future directions: personalized intracranial pressure thresholds

A promising way forward in moderate-severe TBI management is through the development of individualized therapeutic targets, avoiding the current “one treatment fits all” dogma of guideline-based care. A 2002 study by Steiner and colleagues demonstrated that it was possible to calculate personalized optimal CPP targets (CPPopt) by using the relationship between CPP and cerebrovascular reactivity metrics.^119^ The researchers achieved this by plotting CPP against PRx, which revealed a U-shaped curve, and finding the CPP value that most minimized PRx.^119^ Later works have demonstrated the feasibility of continuously calculating such targets for a person, thus making its application to the clinical setting possible.^120,121^ These personalized targets have been reported to have stronger associations with outcome than guideline-based CPP targets.^120,122–125^ For example, Aries and colleagues found that patient outcome, when dichotomized into survival/death, was associated with deviation of CPP from continuously updating CPPopt (*p* < 0.001), and that this association was stronger than that of deviation from the guideline-based CPP target of 60–70 mm Hg.^120^

**FIG. 3.**
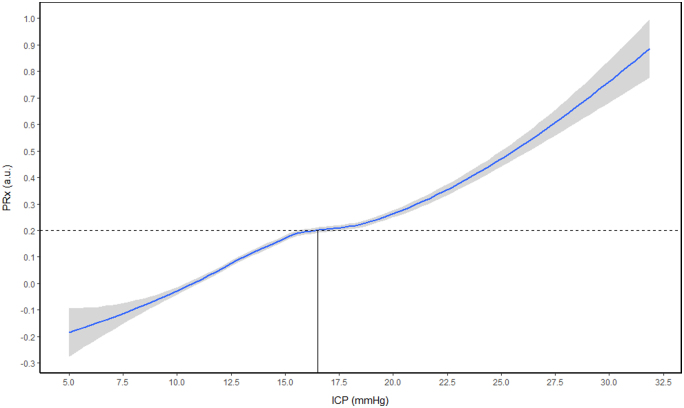
Patient example of iICP generation. LOESS function plot with 95% confidence intervals, intersection with PRx of +0.20 (dotted line) determines the patient's personalized threshold. a.u., arbitrary units; iICP, individualized intracranial pressure threshold; LOESS, locally estimated scatterplot smoothing; PRx, pressure reactivity index. Data from Winnipeg Acute TBI Laboratory Database, with full ethics approval in place for access, processing, and publication (H2017:181, H2017:188, H2020:118).

Despite these promising findings, there has been little work done on personalized ICP thresholds, with only two studies published to date. Preliminary work has demonstrated the presence of such a threshold, defining them as the ICP value past which cerebrovascular autoregulation becomes persistently deranged.^126,127^ Derivation of this individualized ICP threshold (iICP) has been achieved by plotting ICP against PRx and finding the ICP value at which PRx remains above +0.20. A patient example of the generation of iICP is presented in Figure 3. In the study that founded the concept of iICP, Lazaridis and colleagues demonstrated that iICP could be found in ∼68% of patients.^126^ Upon receiver operator curve analysis on predictive power for survival, they found that ICP dose (time × intensity) above iICP had greater discriminative ability over doses >20 or 22 mm Hg, with AUC and 95% confidence intervals of 0.81 (0.74–0.87), 0.75 (0.68–0.81), and 0.77 (0.70–0.83), respectively.^126^ In the second and most recent original work on iICP, Zeiler and colleagues found that iICP could be identified in 65.3% of patients.^127^ On univariate analysis for survival at 6–12 months post-TBI, ICP dose above iICP (AUC = 0.678, *p* = 0.029) was superior to doses above 20 (AUC = 0.509, *p* = 0.03) or 22 mm Hg (AUC = 0.492, *p* = 0.035).^127^

Further work is required to bring this concept closer to clinical utility. First, both studies to date have used the entire recording period for the derivation of iICP. This does not allow for guiding therapeutic management early in a patient's ICU stay, making it impractical for bedside use. Further, the study by Lazaridis and colleagues utilized manual inspection for the derivation of iICP, and though the study by Zeiler and colleagues utilized a semiautomated derivation technique, a continuously updating derivation is needed in order to make such technology even remotely clinically useful. Such continuously updating derivation is likely achievable by using sliding windows of data, similar to those used in the derivation of continuously updating CPPopt.^120^ Future work evaluating the feasibility of such derivation is needed. Second, both studies were only able to identify iICP in ∼67% of patients. In addition to being able to continuously derive iICP, a practical algorithm should also improve on this yield through the management of outliers and missing data. Moreover, work evaluating the relationship between percent yield and various patient factors, such as demographics, injury patterns, and treatment, is needed. Third, with the recent findings on the possible superiority of other cerebrovascular reactivity metrics, such as PAx and RAC over PRx for predicting outcome,^128––131^ further work comparing iICPs derived using the various metrics is needed. Last, additional work evaluating the associations of improved iICP calculations with outcome, that controls for variables known to be associated with outcome, is needed.

## Conclusion

Over the past decades, there have been many innovations in the field of ICP monitoring. This has facilitated the continuous monitoring of ICP at the bedside and its use in guiding therapeutic management. Unfortunately, despite this and the improvements made in our ability to control ICP, there has been very little change in the poor outcomes associated with moderate-severe TBI. This is because of the one-treatment-fits-all approach of current guideline-based care. The concept of iICP is an exciting and promising way forward in moderate/severe TBI management. However, further work validating its presence, automating its continuous calculation, and evaluating its association with long-term outcome in comparison to guideline-based thresholds is still needed.

## Abbreviations Used

<<<AIS: Abbreviated Injury Scale
ABP: arterial blood pressure
AMP: amplitude of the pulse waveform
AUC: area under the curve
BIS: bispectral index
BMI: body mass index
BTF: Brain Trauma Foundation
CBF: cerebral blood flow
CPP: cerebral perfusion pressure
CPPopt: optimal CPP
CSF: cerebrospinal fluid
CT: computed tomography
EtCO_2_: end-tidal carbon dioxide
EVD: external ventricular drain
GCS: Glasgow Coma Scale
ICH: intracranial hypertension
ICP: intracranial pressure
ICU: intensive care unit
MAP: mean arterial pressure
PAx: pulse amplitude index
PbtO_2_: parenchymal brain oxygen tension
PRx: pressure reactivity index
TBI: traumatic brain injury
